# Supplementary material for: Long-Term Treatment with Low-Level Arsenite Induces Aberrant Proliferation and Migration via Redox Rebalance in Human Urothelial Cells
Source: Cells. 2025 Jun 16;14(12):912. doi: 10.3390/cells14120912 (PMC12190571; doi:10.3390/cells14120912)
Supplement: Supplementary file 1 [file cells-14-00912-s001.zip › cells-3662658-supplementary.pdf]

Supplemental Figures

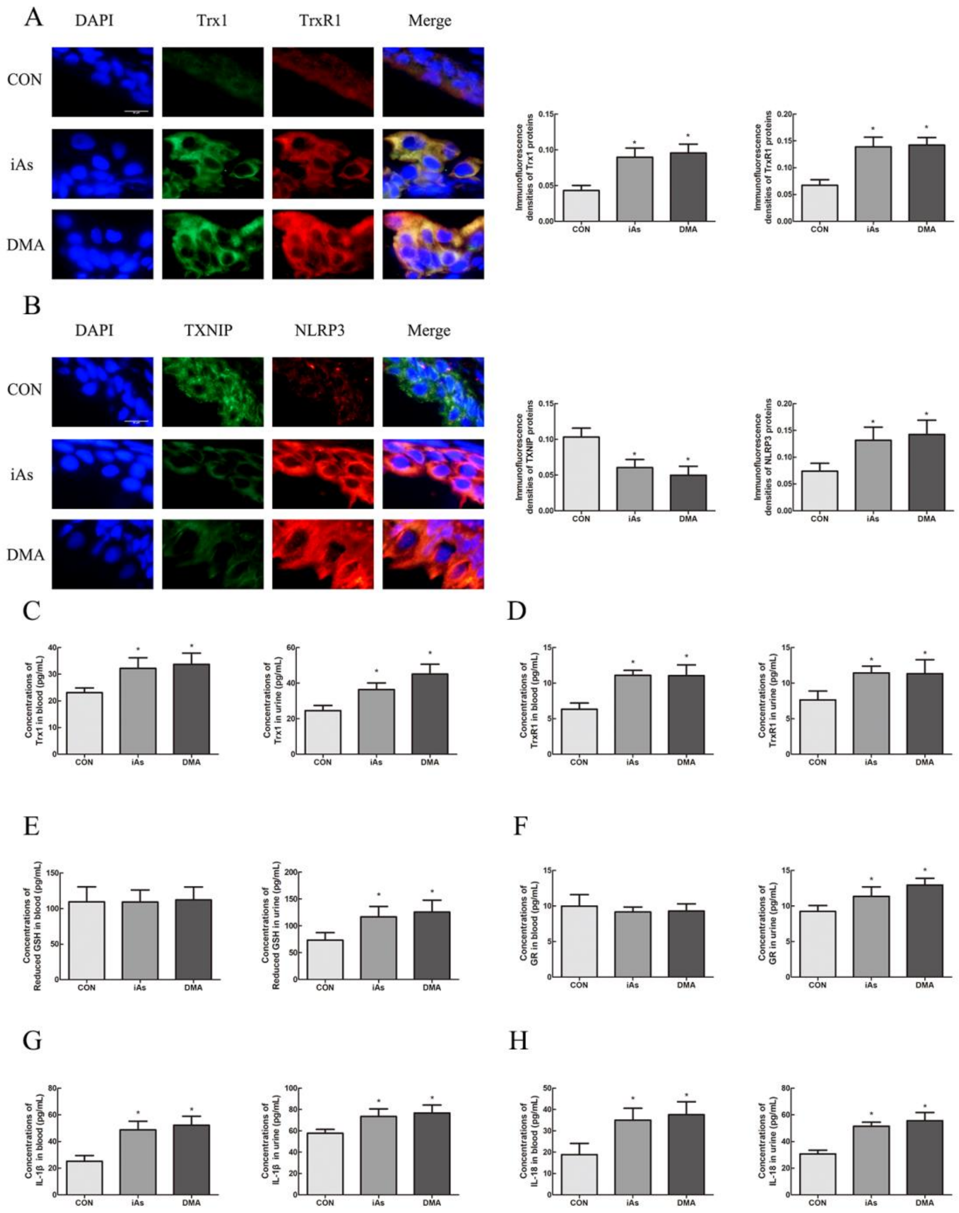

Figure S1. *Protein levels of Trx1, TrxR1, TXNIP, NLRP3, GSH, GR, IL-1β, and IL-18 in rat urothelium treated chronically with arsenite and DMA for 12 weeks.*

Photos and graphs illustrate the immunofluorescence staining and quantification for the protein levels of Trx1 and TrxR1 (A), and TXNIP and NLRP3 (B) in rat urothelium. The graphs show the protein levels of Trx1 (C), TrxR1 (D), and reduced GSH (E), GR (F), and levels of IL-1β (G) and IL-18 (H) in rat blood (on the left panel) and urine (on the right panel) examined by ELISA.

Adult female F344 rats were treated with 50 ppm arsenite or 108 ppm DMA through drinking water for 12 weeks. DMA, dimethylarsinic acid.
